# GARP as a Therapeutic Target for the Modulation of Regulatory T Cells in Cancer and Autoimmunity

**DOI:** 10.3389/fimmu.2022.928450

**Published:** 2022-07-08

**Authors:** Niklas Zimmer, Emily R. Trzeciak, Barbara Graefen, Kazuki Satoh, Andrea Tuettenberg

**Affiliations:** ^1^ Department of Dermatology, University Medical Center Mainz, Johannes Gutenberg University Mainz, Mainz, Germany; ^2^ Early Clinical Development Department, Daiichi Sankyo Co., Ltd., Tokyo, Japan; ^3^ Research Center for Immunotherapy, University Medical Center Mainz, Johannes Gutenberg University Mainz, Mainz, Germany

**Keywords:** LRRC32, GARP mRNA, Glycoprotein A repetitions predominant (GARP), Treg, Soluble GARP or soluble Glycoprotein A repetitions predominant (sGARP), biomarker, therapy

## Abstract

Regulatory T cells (Treg) play a critical role in immune homeostasis by suppressing several aspects of the immune response. Herein, Glycoprotein A repetitions predominant (GARP), the docking receptor for latent transforming growth factor (LTGF-β), which promotes its activation, plays a crucial role in maintaining Treg mediated immune tolerance. After activation, Treg uniquely express GARP on their surfaces. Due to its location and function, GARP may represent an important target for immunotherapeutic approaches, including the inhibition of Treg suppression in cancer or the enhancement of suppression in autoimmunity. In the present review, we will clarify the cellular and molecular regulation of GARP expression not only in human Treg but also in other cells present in the tumor microenvironment. We will also examine the overall roles of GARP in the regulation of the immune system. Furthermore, we will explore potential applications of GARP as a predictive and therapeutic biomarker as well as the targeting of GARP itself in immunotherapeutic approaches.

## Introduction

Regulatory T cells (Treg) play an essential role in the maintenance of immune homeostasis and the induction of peripheral tolerance. They have been shown to suppress many aspects of the immune response by employing multiple immunosuppressive mechanisms. These mechanisms can be broadly classified as being either contact dependent, such as the transfer of cAMP to T effector cells (Teff) *via* gap junctions (1), or contact independent, like the secretion of suppressive cytokines. As a result, many approaches have been tested to target Treg in order to suppress autoimmune diseases or to enhance anti-tumor immunity.

In addition to the transcription factor, Forkhead box P3 (Foxp3), increased expression of Glycoprotein A repetitions predominant (GARP) has been found on the surface of activated Treg. GARP, a transmembrane protein, is the docking receptor for latent transforming growth factor (LTGF-β) and thereby plays a critical role in the production and release of active transforming growth factor beta (TGF-β). TGF-β is a pleiotropic and potent immunosuppressive cytokine known to contribute to both immune modulation and evasion. GARP is also expressed by platelets and by tumor cells of different entities, and it has been detected as a soluble factor. Expression of GARP has been found to be tightly regulated by epigenetic modifications, microRNAs (miRNA), and the master chaperone, GP96, amongst other things.

The objectives of this review are to highlight GARP expression, regulation, and function in Treg, other immune cells, and cancer cells as well as to evaluate the potential of both surface and soluble GARP as predictive and therapeutic biomarkers. In addition, approaches that target GARP for immunotherapeutic intervention in autoimmune diseases and cancer will be discussed.

## GARP Expression, Structure, and Function

### LRRC32 Structure

The gene encoding human GARP can be referred to by various names including: LRRC32, Glycoprotein A Repetitions Predominant, GARP, Transforming Growth Factor Beta Activator LRRC32, Leucine-Rich Repeat-Containing Protein 32, Garpin, CPPRDD, and D11S833E (Gene ID: 2615). For the sake of clarity, we will specifically refer to the GARP gene as “LRRC32*”*, its mRNA as “GARP mRNA”, and the protein as “GARP” for the remainder of the manuscript.

LRRC32 was first described in the telomeric region of 11q13.5-11q14 in human (2) and mice (3). LRRC32 consists of two exons (4), and its expression is conferred by two alternative promoters (5). One exon codes for a signal peptide as well as nine amino acids, while the other codes for leucine-rich repeats (LRR). In addition, LRRC32 contains an extensive 2-kb long 3′ untranslated region (UTR), that has five highly conserved regions which are of importance for the post-transcriptional regulation of the GARP mRNA (6).

So far, the GARP mRNA has been detected in various cell types and tissues of different origin, including heart, kidney, liver, lung, pancreas, placenta, skeletal muscle, and lymphoid tissues as well as in different cancer entities (e.g. melanoma, breast cancer, oral squamous cell carcinoma, prostate cancer, and glioblastoma). Although the GARP mRNA is expressed by many cell types, surface expression of the GARP protein itself has been only reported in the context of activated Treg (7, 8), activated B cells (9, 10), macrophages (11), platelets (12), mesenchymal stem cells (13), and hepatic stellate cells (9, 14). In Treg, surface GARP is considered to be an activation marker. Of note, the GARP mRNA is also expressed by human Teff clones. Nevertheless, even though its expression levels in such clones are similar to levels found in some Treg clones, GARP has not been detected on the surface of either human or mouse activated Teff (15).

Interestingly, the LRRC32 gene locus is part of a chromosomal region that was described to be altered in several human cancers. In agreement with this finding, the GARP mRNA is highly amplified in tumor cells, and GARP surface expression has been detected in invasive, metastatic, and drug resistant tumors (16–18). Furthermore, in ovarian cancer, single nucleotide polymorphisms (SNP) were described in the non-coding regions of the LRRC32 (two were found in the 3’ UTR, and one was found in the intron (rs3781699 and rs7944357, respectively), which have been associated with poor patient survival (19). Additionally, the gene locus of LRRC32 was also identified as a risk locus for asthma (20), atopic dermatitis (21), and colitis (22).

### GARP Structure

GARP is an approximately 78 kDa type I transmembrane protein made-up of 662 amino acids with an extracellular region consisting of 20 LRR. In more detail, its structure has three domains: a cytoplasmic tail of 15 amino acid residues, a hydrophobic transmembrane domain, and an extracellular domain, containing the LRR, which accounts for about 70% of the protein (2). In addition, a signal peptide is located in the N-terminus, and its cleavage is required for the surface expression of GARP (4). The extracellular domain of GARP is similar to the corresponding region of other members of the LRR protein family, which in general play an important role in protein-protein interactions and signal transduction (2). It contains 20 LRR motifs, subdivided into two groups of 10 LRRs each by a proline rich domain and a C-terminal LRR [20]. The proline rich region confers flexibility and supports the idea of the involvement of GARP in protein-protein interactions. The two cysteines, Cys-192 and Cys-331, located in the 7th and 12th LRR respectively, are responsible for the two disulfide bonds that form between GARP and its ligand, latency associated peptide (LAP), in the LTGF-β complex (23). Following translation, GARP undergoes N-linked glycosylation and contains five predicted glycosylation sites (2, 24).

### GARP Function

TGF-β is a pleiotropic cytokine, that is an important mediator during the development of Treg and the maintenance of their immunoregulatory state (25). Besides Treg, TGF-β is expressed by a multitude of cell types and tissues and participates in the mediation of numerous pathways, including development, wound healing, homeostasis, and cancer (26).

**Figure 1:**
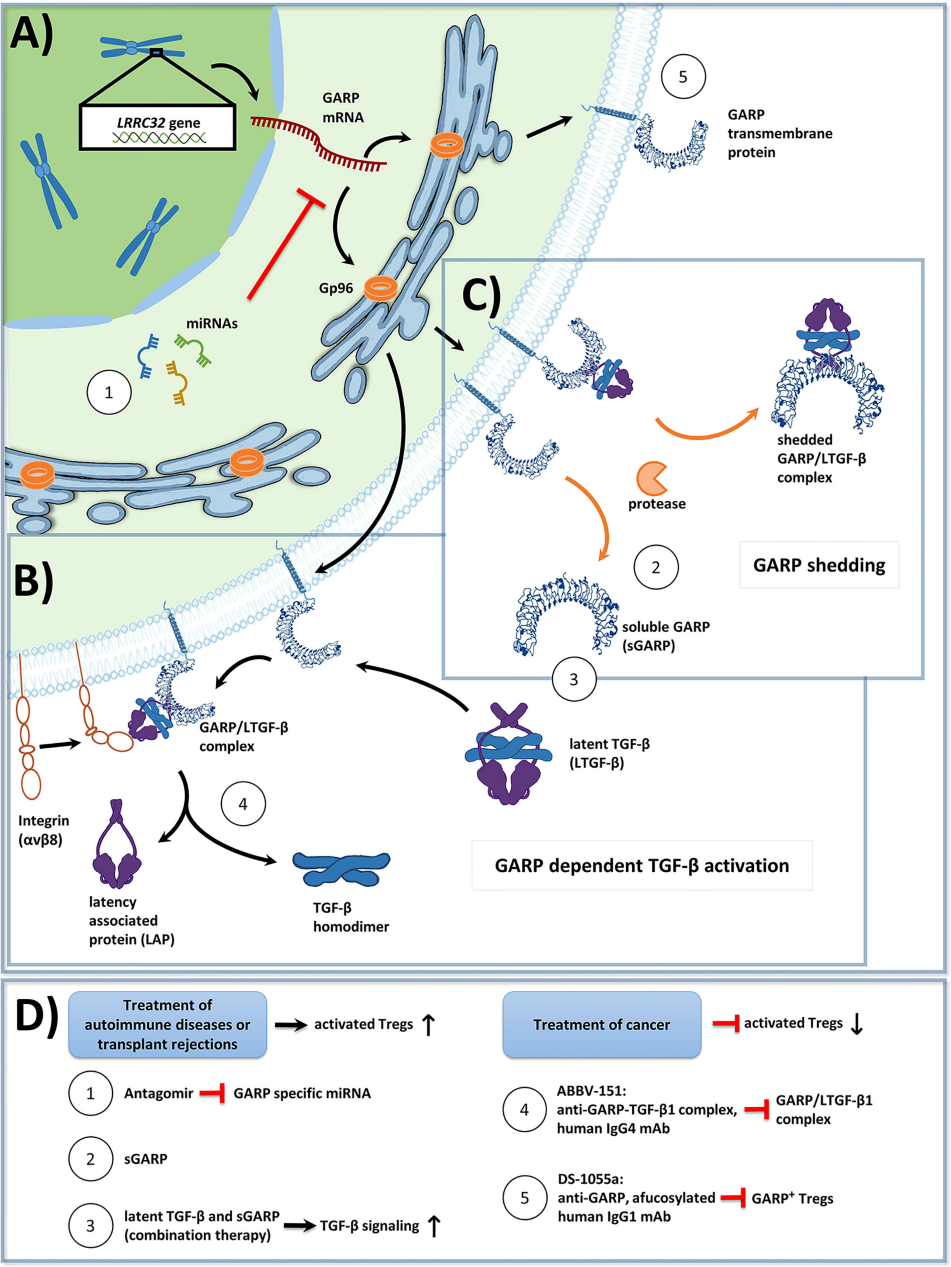
**(A)** Overview of Glycoprotein a repetitions predominant (GARP) protein biosynthesis and transport to the cell membrane in activated human regulatory T cells (Tregs). GP96, a chaperone found in the endoplasmic reticulum, ensures proper folding of GARP [GARP structure modified from: Liénart et al. (35)]. The GARP mRNA is targeted by miRNA, which promote its degradation, and thus lower GARP mRNA/GARP levels. **(B)** GARP functions as a docking receptor for biologically inactive latent transforming growth factor beta (LTGF-β), which consists of a TGF-β homodimer bound to latency associated protein (LAP), and GARP plays an important role in its activation. GARP binds LTGF-β with high affinity, forming the GARP/LTGF-β complex. Release of bioactive TGF-β can occur in both a protease independent (shown) or a protease dependent manner (not shown). For protease independent release of mature TGF-β, α_V_β_8_ integrins, expressed on the surface of Treg bind to the GARP/LTGF-β complex, resulting in a conformational change and in the subsequent release of biologically active TGF-β. Alternatively, bioactive TGF-β can be released in a protease dependent manner, in which integrin recruited metalloproteinases or serine proteases cleave LAP from the GARP/LTGF-β complex (not shown). **(C)** GARP can be cleaved from the surface of Treg by proteases in a form called soluble GARP (sGARP). GARP/LTGF-β complexes can also be released into the extracellular environment via proteases. **(D)** Potential methods to target GARP for the treatment of autoimmune diseases, transplant rejections, and cancer. Enhancing GARP mediated suppression by Treg offers a promising strategy for the treatment of autoimmune diseases and transplant rejections. (1) Approach 1 utilizes antagomirs, specific to miRNA, which target the GARP mRNA. This would prevent GARP mRNA degradation, and thus increase surface GARP expression and enhance the suppressive capacity of Treg. (2) Approach 2 is to apply sGARP to induce Treg. (3) Approach 3 would be to apply sGARP in combination with LTGF-β to harness both their immunosuppressive effects and to promote the activation of TGF-β in an integrin-controlled manner. For effective anti-tumor immune responses to occur, Treg mediated suppression needs to be inhibited. (4-5) Approaches 4 and 5 represent two different monoclonal antibody (mAb) therapeutic strategies, that are currently in phase 1 clinical trials. (4) Approach 4 uses an IgG4 antibody (ABBV-151) that binds to the GARP/TGF-β1 complex and prevents the release of mature TGF-β1. This results in an inhibition of TGF-β1 signaling, a subsequent decrease in the suppressive capacity of Treg, and the restoration of T effector cell (Teff) functions. (5) Approach 5 employs an afucosylated IgG1 antibody (DS-1055a), that efficiently depletes GARP^+^ Treg via antibody-dependent cellular cytotoxicity, preventing Treg mediated suppression and restoring Teff function.

GARP has been shown to be essential for the formation and surface expression of LTGF-β on Treg. GARP binds all three isoforms of TGF-β (7, 27, 28) and plays an important role in TGF-β activation, which is first synthesized as a biologically inactive homodimeric precursor protein (23, 29, 30). This proprotein consists of three distinct parts: (I) mature TGF-β, (II) LAP, and (III) a signal peptide. Following the removal of the signal peptide, *via* cleavage by furin proteases, inactive TGF-β becomes mature TGF-β (31). Then, mature TGF-β binds LAP through both covalent (disulfide bridges) and non-covalent interactions (32). The resulting complex of mature TGF-β and LAP is called latent TGF-β (LTGF-β) and lacks biological activity. In the absence of GARP, LTGF-β binds to the latent TGF-β binding protein (LTBP), thereby forming the large latent complex (LLC), which associates with the extracellular matrix (ECM) (33). Surface GARP inhibits the binding of LTGF-β to the LTBP due to its higher affinity and in turn, presents LTGF-β on the cell surface. GARP enables the binding of latent TGF-β to α_V_β_6_ and α_V_β_8_ integrins, forming a ring-like shape with TGF-β orientated towards the center. This enables the release of TGF-β from LTGF-β mediated by a protease dependent or a protease independent mechanism. Integrin recruited metalloproteinases or serine proteases may cleave LAP from the TGF-β – LAP - GARP complex. Of note, α_V_β_6_ and α_V_β_8_ are expressed in a cell type specific manner. In particular, Treg express α_V_β_8_ (34). Protease independent release of TGF-β is facilitated through the binding of the respective integrins to LTGF-β and the resulting deformation of LAP, triggered by cell contraction. This results in the release of bioactive mature TGF-β into the extracellular space (34) (**Figure 1B**).

## GARP Regulation GARP and Its Regulation in Treg

### Interplay of GARP and Foxp3

The detailed relationship between Foxp3 and GARP in Treg remains a matter of debate.

At first, it was described that the regulation of GARP is independent of Foxp3. This conclusion was based on studies, which demonstrated that TGF-β induced overexpression of Foxp3 was not sufficient enough to induce the expression of GARP (7, 36). Furthermore, it was shown that the knockout of Foxp3 in Treg did not change GARP expression, and correspondingly, the knockdown of GARP did not affect Foxp3 expression. However, a knockdown of GARP led to an impaired suppressive capacity of Treg, while silencing of Foxp3 in GARP-expressing cells did not affect their suppressive capacity, but knockdown of Foxp3 lead to an impaired Treg function (8). Interestingly, GARP is not expressed by resting CD4^+^Foxp3^+^ Treg, but it is upregulated upon Treg activation. In comparison, CD4^+^Helios^+^Foxp3^-^ cells upregulate the expression of GARP/LAP upon TCR stimulation, supporting that Foxp3 and GARP are not regulated by each other (37).

In contrast to this conclusion, Probst-Kepper et al. proposed a mutual dependency of Foxp3 and GARP expression, which occurs in a positive feedback loop like manner. They were able to demonstrate that lentiviral downregulation of the GARP mRNA led to the downregulation of Foxp3 and the loss of Treg suppressive properties. Similarly, the downregulation of Foxp3 also resulted in the downregulation of GARP mRNA and impaired suppressive Treg function (15).

### Pre-Transcriptional Regulation of LRRC32

Haupt et al. improved our understanding of the regulation of LRRC32 by showing that the transcription factors, nuclear factor of activated T cells (NFAT) and nuclear factor kappa light chain enhancer of activated B cells (NF-κB), play an important role in the expression of LRRC32. Transcription of LRRC32 is driven by two different promoter regions, P1 and P2, which differ in their methylation status depending on the cell type examined and the surrounding environmental conditions. It was shown, that in Treg, P1 and P2 are completely demethylated. This allows Foxp3 to bind to P1, opening the promoter region *via* chromatin remodeling, enabling the binding of NFAT and NF-κB, resulting in the expression of LRRC32. In contrast, Th cells differ from Treg as they exhibit increased methylation of their P1 promotor, which consequently prevents the expression of LRRC32 (5).

In 2020, Nasrallah et al. were able to show that an enhancer, located at chromosome 11q13.5, is active in Treg (22). This enhancer forms conformational interactions with the promoter of LRRC32, and the enhancer risk variant, rs11236797, is associated with a reduction in histone acetylation and decreased LRRC32 expression. This is based on the recruitment of STAT5 and NF-κB (22), which in turn mediate the expression of LRRC32. Therefore, these transcription factors are vital for Treg-mediated suppression. Any disruption of LRRC32 leads to early lethality in mice. The knockout of the enhancer led to the development of Treg that did not express LRRC32/GARP, which were unable to control colitis in an adoptive transfer model. This underlines the results of previous studies, that indicated that GARP is necessary for Treg function (8, 22).

Recently, Lehmkuhl et al. demonstrated that GARP-deficient murine Tregs were characterized by an unstable Treg phenotype as reflected by the decreased expression of CD25, Neuropilin-1(Nrp1), cytotoxic T-lymphocyte-associated Protein 4 (CTLA-4), Interleukin-10 (IL-10), and Histone deacetylase 9 (HDAC9), and they were characterized by impaired immunosuppressive activity (38). These alterations were due to decreased acetylation of Foxp3 in comparison to stable Treg. Interestingly, it was found that GARP could regulate the expression of HDAC9, which is responsible for Foxp3 acetylation and thus Treg stability (38, 39). Future studies are necessary to elucidate how HDAC9, NFAT, and NF-κB interact and how their interplay affects the expression of LRRC32/GARP in more detail.

### Post-Transcriptional Regulation of the GARP mRNA

It is becoming increasingly apparent that the expression of GARP is tightly regulated. Hereby, miRNA have been shown to play an important role. miRNA are single stranded, conserved, non-coding RNA molecules that play an essential role in post-transcriptional regulation (40). By binding to sequence complementary sites within the 3’ UTR of their target mRNAs, miRNA lead to translation inhibition and mRNA degradation, which collectively leads to the suppression of gene expression. Numerous miRNA have already been found to regulate the development, differentiation, proliferation, and suppressive function of Tregs, including the targeting of the GARP mRNA itself (41).

As mentioned in *section 2.1, LRRC32 Structure*, the 3’ UTR of LRRC32 contains five evolutionary conserved regions, which are a promising indicator for potential miRNA recognition sites (6). So far, several miRNA have been found to target the 3’ UTR of the GARP mRNA in human Treg. Zhou et al. found that miR-142-3p directly binds to the 3’ UTR of the GARP mRNA and promotes its subsequent degradation *via* the Argonaute 2 pathway in primary CD4^+^CD25^+^ human T cells (6). They could also show that miR-142-3p controls the expression of GARP mRNA/GARP in activated Treg. Upon Treg activation, GARP mRNA/GARP levels are upregulated. However, the reason GARP levels subsequently decline following activation results from the upregulation of miR-142-3p, which binds to the GARP mRNA and targets it for subsequent degradation. Gauthy et al. could show that miR-185, and miR-181 a, b, c, d, in addition to miR-142-3p, target the 3’ UTR of GARP mRNA in human Treg differentiated from PBMCs (31) (**Figure 1A**). Furthermore, they could show that these miRNA were also expressed at a lower level in human Treg when compared to Th cells. Interestingly, a study by Jebbawi et al. demonstrated that miR-24 and miR-335 directly bind to the 3’ UTR of the GARP mRNA and regulate GARP levels (34). It is important to note that they examined primary human CD8^+^CD25^+^ Treg derived from cord blood in contrast to the aforementioned studies, which studied human CD4^+^ Treg (42). These differences in cell type and tissue source may help explain in part why different sets of miRNA were detected.

### Post-Translational Regulation of GARP

Surface expression of GARP has been found to be dependent on the heat shock protein, GP96 (GRP94) (43). GP96 is a master chaperone, which is found in the endoplasmic reticulum (ER), and its clientele consists of proteins implicated in both immune response and oncogenesis, such as toll-like receptors (TLR), integrins, Wnt co-receptor low-density lipoprotein receptor-related protein 6 (LRP6), insulin-like growth factor (IGF), platelet glycoprotein Ib-IX-V complex, and human epidermal growth factor receptor-2 (HER2) (43, 44). In a study by Zhang et al., examining the effects of GP96 knockout (KO) in Treg *in vivo*, it was discovered that although GP96 KO mice developed Treg, they displayed unstable Foxp3 expression, increased IFN-γ production, and impaired suppressive function, thus leading to the development of fatal autoimmunity (43). One reason for this reduction in Treg suppressive function was a decrease in surface GARP expression and mature LTGF-β levels. Loss of surface GARP and mature LTGF-β was also observed in CD41^+^ GP96 KO platelets and GP96 deficient B cells, suggesting that GP96 acts as an obligate chaperone for GARP. Loss of surface GARP expression in GP96 deficient B cells was attributed to the inability of GARP to leave the ER and was accompanied by a decrease in the half-life of GARP. Collectively, this suggests that GP96 is required for the stable protein conformation of GARP. In addition, it was discovered that GARP interacts with the C-terminal client-binding domain of GP96 as also reported for TLRs (43).

Another protein that interacts with GARP, lysosomal associated transmembrane 4B (LAPTM4B), was identified in a yeast two hybrid assay (37). LAPTM4B expression increased upon Treg activation, and it directly decreased the surface expression of GARP and the secretion of LTGF-β1. LAPTM4B has been postulated to function as part of a negative feedback mechanism to downregulate Treg production of LTGF-β1 and surface GARP during T cell activation. Translocation of intracellular GARP to the surface of Treg upon activation requires the cleavage of a signal peptide located in the N-terminus of GARP (4). It has also been found in a model of forced GARP overexpression in T cells, TCR activation was needed for the translocation of intracellular GARP to the cellular surface. Furthermore, in case of Treg, IL-2 signaling is able to specifically increase the surface expression of GARP (45, 46).

It has been described that activated Treg shed a soluble form of GARP (sGARP) from their surfaces (18). Shedding of GARP was first proposed by Roubin et al. in their description of the protein structure of GARP. They hypothesized, that a hydrophobic leader sequence embedded in the amino acid sequence of GARP may resemble a signal peptide for secretion (3). The shedding of GARP has been subsequently confirmed in blood plasma by mass spectrometry (47). Additionally, Metelli et al., were able to show that thrombin can cleave GARP from the surface of platelets, which is essential for the release of membrane latent TGF-β (mLTGF-β) (48). Shedding of GARP/LTGF-β1 complexes from the surfaces of stimulated Treg and GARP overexpressing Th cells has also been described and detected in cell supernatants (31) (**Figure 1C**). These complexes may have been shed from the membrane by proteases, and their functional significance remains unknown.

## Soluble GARP

As mentioned earlier, sGARP is released by an array of different cell types, including activated Treg, activated platelets, and cancer cells. Hahn et al. could show that sGARP can modulate immune responses and has strong suppressive properties (49). In this regard, recombinant sGARP was found to suppress the proliferation and cytokine production of Teff. Exposure of naive T cells to sGARP led to an induction of Treg. This transition was accompanied by the induction of Foxp3 expression, the inhibition of cell proliferation, and a significant decrease in IL-2 and IFN-γ production. Furthermore, sGARP induced a tumor associated “M2-like” macrophage (TAM) phenotype and suppressed cytotoxic T cell function by inhibiting cell proliferation and the production of IFN-γ and Granzyme B (49).

Additionally, in a humanized mouse model of a xenogenic graft versus host disease (GvHD), the application of recombinant sGARP protected the animals from T cell mediated inflammation through Treg activation (49). It was also found that sGARP drives epithelial-mesenchymal cell transition (EMT). Cells treated with sGARP showed increased proliferation and migratory capacities in comparison to the untreated control (30).

The mechanism by which sGARP induces these phenotypic changes is at least in part dependent on the TGF-β signaling pathway, as phosphorylation of Mothers against decapentaplegic homolog 2 and 3 (Smad2/3) in sGARP treated naive CD4^+^ T cells was observed. However, inhibition of TGF-β signaling by the use of a TGF-β receptor II blocking antibody could not fully prevent the effects of sGARP (49).

These findings could be confirmed by our group using a physiological source of sGARP (12). Activated platelets have been shown to shed GARP from their surfaces. Based on these findings, CD4^+^ T cells were cocultured with platelet conditioned medium (PCM). PCM was able to induce Treg, characterized by a strong Foxp3 expression, while simultaneously suppressing their proliferation as well as their IL-2 and IFN-γ production when compared to the untreated control. Administration of a blocking anti-GARP antibody was able to mitigate these effects. Furthermore, it was shown that blockade of the TGF-β pathway, by applying blocking antibodies against TGF-β I-III and TGF-β RII, could not completely inhibit the effects of PCM on T cells. This indicates that sGARP mediates its effects in part through a TGF-β independent signaling pathway (12).

Furthermore, it has been shown that recombinant sGARP has a strong capability to enhance the activation of free latent TGF-β. This function further amplifies the effects of sGARP in the case of autoimmunity and cancer (50).

Collectively, this leads to the conclusion that sGARP is able to achieve two things in parallel, which may multiply its immunomodulatory effects. First, sGARP has the ability to modulate the differentiation and suppression of immune cells. Second, sGARP can enhance the activation of TGF-β and correspondingly its manifold downstream effects. Therefore, sGARP may act as an important player in both autoimmunity and the tumor microenvironment (TME). Taken together, this highlights the importance of sGARP as a potent immunoregulatory molecule on its own and in its interplay with TGF-β.

## Cellular GARP

### Regulatory T Cells

CD4^+^CD25^+^CD127^low^Foxp3^+^ Treg, a highly immunosuppressive subset of CD4^+^ T cells, play a major role in immune homeostasis by controlling immune responses through the induction and maintenance of peripheral tolerance (51). As mentioned above, GARP is expressed on the surface of activated Treg and plays a vital role in conveying their suppressive capacity (**Figure 1A**). GARP expression is also obligatory for the binding of TGF-β1 to the surface of Treg (7, 36).

Until recently, the functions of GARP on Treg were described as aiding in the presentation of LTGF-β by acting as both an anchor and support protein for the activation and release of LTGF-β (7). It has been shown that GARP^+^ Treg in comparison to GARP^-^ Treg displayed a greater suppressive capacity of Teff *in vitro*. This difference was associated with a decrease in the effector cytokines IL-2 and IFN-γ production (15, 36) and a corresponding increase in the production of the inhibitory cytokines TGF-β and IL-10 (52, 53).

Resting murine Treg express a low level of GARP. Upon TCR stimulation GARP gets upregulated on the cell surface of Treg, followed by an increased expression of latent TGF-β1. GARP expression can be upregulated on murine Treg *via* exposure to IL-2 and IL-4 without the need for TCR activation *in vitro*. Expression of GARP in mice was found to be independent of TGF-β1, but specific KO of GARP in murine CD4^+^ T cells leads to a diminished expression of TGF-β1 on the surface of activated Treg. These GARP^-^ Treg were found to develop normally and were capable of suppressing Teff *in vitro*. Additionally, Treg numbers in the periphery were not affected. Treg which express GARP/LTGF-β1 on their surfaces are able to induce Treg in the presence of IL-2, while the presence of IL-6 leads to the induction of Th17 cells (45, 46).

Further evidence highlighted the importance of GARP in Treg function. In a humanized mouse model of allogenic graft rejection, CD4^+^CD25^high^CD127^low^ Treg and CD4^+^CD25^high^ Treg showed a significantly lower capacity in preventing alloreactions in comparison to CD4^+^CD154^−^GARP^+^ and CD4^+^CD154^−^LAP^+^ Treg (54). This study concluded that LAP and GARP are specific markers for human Treg having a high suppressive activity. In addition, a complete depletion of activated GARP^+^ Treg in a humanized mouse model of allergen-induced gut inflammation diminished the protective effects of Treg (55).

In Treg with mutated LRRC32, expression of GARP was reduced, resulting in an unstable Treg phenotype that led to severe immune dysregulation and an increased development of inflammatory diseases (38). In giant cell arteritis, the most common primary arteritis, based on an imbalance of activated Teff cells and dysfunctional Treg, patients’ Treg showed an ineffective and reduced induction of GARP (56).

In a preclinical approach, *in vitro* expanded Treg were isolated based on their selective surface expression of LAP. Herein, LAP^+^GARP^+^Foxp3^+^ Treg showed a highly demethylated Treg-Specific Demethylated Region, indicating a stable Foxp3 expression and ultimately a stable Treg phenotype. Additionally, these cells showed a high suppressive capacity *in vitro* and in a GvHD *in vivo* model, making these cells a suitable population for the treatment of GvHD in patients (57).

Taken together, GARP expression on Treg significantly influences the immunological balance in different settings: GARP^+^ Treg lead to immunosuppression, being of importance especially in the tumor microenvironment. Deficiency of GARP in Treg has an impact on the development of inflammatory diseases including autoimmunity, allergy and transplant rejection.

### Non-Treg Cells

Besides Treg, B cells are known to express GARP when activated by TLR ligands, such as TLR4, TLR7, and TLR9 (58). Expression of GARP led to the inhibition of cell proliferation, induced a class switch to IgA production, and resulted in a more tolerogenic B cell phenotype. This has been explored especially in the context of autoimmune diseases (10, 58). Herein, GARP was upregulated on B cells in autoimmune diseases and the GARP-LTGF-β axis was shown to be an important factor for B cell tolerance and prevention of lupus-like autoimmune diseases in mice.

Furthermore, hepatic stellate cells constitutively express GARP on their surfaces. A blockade or knockdown of GARP resulted in an impaired suppression of T cell proliferation and IFN-γ production. It has been described that GARP is required to anchor and activate LTGF-β. Whether the observed effects are mainly mediated by LTGF-β, a suppressive function of GARP itself, or by a release of sGARP has not yet been described (14). Nevertheless, being expressed on cells involved in fibrosis as well as in hepatic cell cancer progression, it will be of interest to further investigate influence of GARP in this context.

In multipotent mesenchymal stromal cells (MSC), GARP has been shown to be important for their proliferation and survival by rendering them more resistant to DNA damage and apoptosis in a TGF-β dependent manner (59). In addition, GARP is involved in the immunomodulatory activities of MSC (60).

### Platelets

Expression of GARP was initially described on the surface of platelets (3). Platelets are the main cells that mediate hemostasis at the site of injury. Platelets are important modulators of both innate and adaptive immunity through their interaction with immune cells. In the case of infection, platelets become activated and are able to modulate inflammation (61, 62). In addition, recent evidence indicates that platelets are present in the TME, and cancer associated thrombocytosis has been associated with the promotion of invasion and metastasis, and thus poor clinical outcomes in different tumor entities (63). Low platelet counts and inhibited platelet activation in patient blood correlated with a lower likelihood of metastasis (64, 65). In more detail, platelets promote motility (66–68) and EMT (69). Furthermore, platelet count and activation status influenced the survival of circulating tumor cells (CTC) by shielding them from NK cells and from destruction by shear stress (70, 71). This is due to their expression of fibrinogen receptor GPIIb-IIIa and P-Selectin, which mediate the attachment of platelets onto CTCs *via* the binding of CD44 and avB3 integrin (70). In addition, melanoma cells express chemokines that attract and activate platelets, in a process called tumor cell-induced platelet aggregation (72), resulting in the shielding of metastasizing melanoma cells by platelets in the bloodstream. Platelet-derived TGF-β has been shown to be an important modulator of the immune system (73). Besides being known for its immunosuppressive capability, TGF-β can downregulate B-cell lymphoma 2 (Bcl-2) proteins, known for their anti-apoptotic effects, as well as natural killer group 2D (NKG2D) leading to a decrease in NK cell efficacy (74). While there is increasing evidence that GARP on platelets plays an important role in immunomodulation, GARP does not seem to play a significant role in hemostasis and thrombosis. The conditional knockout of GARP on platelets and endothelial cells in mice did not lead to any changes during agonist induced platelet activation and aggregation. Furthermore, the tail bleeding time and the FeCl3-induced thrombus formation occlusion time were not affected (75).

There has been an emerging role of platelets not only in hemostasis but also in the immunomodulation of cancer patients. Given the fact that platelets express GARP on their surfaces and connecting this to previous works describing GARP as a key molecule in inducing peripheral tolerance, several groups have shown a possible contribution of platelets to adaptive immunity, leading to a poor prognosis of cancer patients with cancer associated thrombocytosis. For example, GARP was found to be expressed on platelets to a certain extent; however, upon platelet activation, surface GARP levels were found to be significantly increased (12). We demonstrated that, cocultures of platelets and Teff as well as Teff grown in the presence of PCM induced a regulatory phenotype, characterized by an upregulation of Foxp3, reduced proliferation, and decreased effector cytokine production, as well as an induction of suppressive capacity. This phenomenon was shown to be GARP dependent. In addition, the TGF-β signaling axis seemed to be at least in part associated to GARP mediated Treg induction (12). The cleavage of GARP by proteolysis through thrombin from the surface of platelets, has been shown to be a major contributor to cancer immune evasion. The blockade of the GARP cleavage led to an improved therapeutic efficacy of anti-PD-1 therapy (48). Furthermore, activated platelets released sGARP, as shown by us and Metelli et al., which in turn induces Treg in a GARP dependent manner (12, 48).

### Tumor Cells

As already mentioned, GARP has been shown to be expressed by several tumor entities, like malignant melanoma (18), glioblastoma (16), bone sarcoma (17), breast cancer (76), and lung cancer (77).

It has been shown that the relation between Foxp3, GARP, and TGF-β, as thoroughly analyzed in Treg, also plays an important role in cancer progression. Tumor cells employ a form of “Treg mimicry”, by utilizing specific immunosuppressive strategies, similar to Treg, to modulate their surroundings (16, 18, 78, 79). In bone sarcoma, GARP plays a vital role in cancer cell proliferation and resistance against irradiation and chemotherapy. Silencing of GARP in these cells led to a decrease in cell proliferation and an increase in apoptosis (17). Li et al., were able to show that GARP also plays an important role in the regulation of TGF-β1 in osteoblast differentiation. Downregulation of the GARP mRNA/GARP in bone marrow mesenchymal stem cells (BMSC) attenuated their differentiation into osteoblasts (80). Furthermore, normal murine mammary epithelial cells, showed an increased production of TGF-β and oncogenesis when GARP was overexpressed. Furthermore, the murine mammary gland tissue cell line, NMuMG, that was unable to form tumors *in vivo*, was able to do so once GARP was overexpressed (30).

In a coculture with CD4^+^ T cells, glioblastoma cells were able to suppress the proliferation and IFN-γ production of the former. By using a blocking anti-GARP antibody, the proliferation and cytokine production of CD4^+^ T cells could be restored (16). Malignant melanoma has been shown to release sGARP into its surroundings, suggesting a further contribution to a GARP-TGF-β mediated immunosuppressive microenvironment (18).

Zhang et al. showed that in pancreatic cancer, tumor cells were able to reprogram M1-like macrophages metabolically and functionally through a GARP-dependent and *via* a DNA methylation-mediated mechanism to M2 macrophages with a pro-tumorigenic phenotype (11).

All these data show that GARP on tumor cells is I) maybe involved in tumor cell proliferation and II) significantly modulates immune responses leading to an inhibitory tumor micromilieu, both facts resulting in massive tumor promotion.

## Role of GARP in Disease Settings

The presence of GARP as a soluble factor and as a surface marker on different cell types influences immune mediated diseases, such as cancer, allergy, and autoimmunity. The presence of GARP, e.g. in tumors, leads to a suppression of immune responses, whereas the loss of GARP, e.g. in autoimmunity, leads to spontaneous inflammation. As such, GARP can be used as both, a biomarker and therapeutic target.

### GARP as a Biomarker

Examining sGARP levels in easily accessible specimens, like serum and plasma from routine blood tests, and correlating their content to patient diagnoses and clinical outcomes is a novel approach to evaluate the potential of sGARP as a diagnostic and prognostic biomarker. This information would be highly valuable for clinicians as it is not only a more practical, time efficient, and cost-effective approach than screening for GARP expressing cells, but it is also a less invasive procedure for patients. Reliable assessment of the concentration of sGARP in patient blood could be of high importance as even low levels of sGARP are capable of greatly enhancing the activation of TGF-β (50). Therefore, elevated sGARP levels in cancer patients could have a great impact on the TME, leading to unfavorable patient outcomes.

Metelli et al., were able to detect high levels of sGARP and sGARP/LTGF-β1 complexes in the blood plasma of prostate cancer patients. Increased amounts of sGARP correlated with increased likelihood of metastasis in these patients (48).

Some studies have indicated that TGF-β, which is regulated by GARP, plays an important role in DNA repair, and may protect cells from ionizing radiation. Kim et al., could show that pretreatment of human epidermoid carcinoma cells with TGF-β reduced γ-radiation induced apoptosis (73). However, to the best of our knowledge, no known study has examined the possibility of GARP as a predictive marker for radiation sensitivity in cancer cells yet.

GARP is an enticing potential diagnostic biomarker for cancer as it is highly upregulated on the surface of both tumor cells and suppressive immune cells found in the TME (16, 18). Nevertheless, only a few studies have examined the possibility of GARP as a diagnostic biomarker in further detail. One study by Jin et al., examined the potential of GARP on Treg as a diagnostic biomarker in both the tumor tissue and peripheral blood of lung cancer patients. They found that frequencies of GARP expressing Treg were higher in the tumor tissue of early stage (I-II) versus late stage (III- IV) patients but not in their peripheral blood (77). Additionally, we could show that melanoma patients, who do not respond to immunotherapy, showed a longer overall survival, when they had a low percentage of GARP^+^ Treg (81). This was also the case in hepatocellular carcinoma, as a high frequency of Foxp3^+^GARP^+^ Treg correlated with a more aggressive phenotype and a TME with enhanced suppressive properties (82).

A study by Metelli et al. showed that high GARP expression in lung cancer versus healthy adjacent tissue correlated with a reduced overall survival (30). The same observation was also described in bone sarcoma (17). Furthermore, it has been shown that GARP expression is increased in gastric cancer (83). Here, increased levels of CD4^+^GARP^+^ T cells in the tumor vicinity correlated with poor overall survival. Interestingly, elevated expression of GARP also correlated with the expression of CTLA-4 and PD-L1 (83).

Correlation data listed by the Human Protein Atlas, based on the TCGA data set, show that renal and urothelial cancer patients with elevated GARP mRNA levels have a significantly lower overall survival when compared to patients with lower GARP mRNA concentrations (proteinatlas.org/ENSG00000137507-LRRC32). This was not observed though for human lung cancer.

As mentioned before, it has been described that GARP expression was increased on the platelets of melanoma patients, but there were no differences between stage I and stage IV patients (12). One potential limitation of this study was the composition of the patient cohort, as 15 out of 16 patients responded to immunotherapy, potentially impacting the GARP expression levels found on platelets. Nevertheless, a potential use of GARP as a biomarker on platelets should be analyzed in a larger patient cohort (12). As outlined above, an increased platelet count in malignant melanoma patients is associated with poor prognosis. Furthermore, patients, who did not respond to immunotherapy, had an increased platelet to lymphocyte ratio as compared to responders to immunotherapy (12, 81, 84). Elevated platelet counts, increased expression of GARP on platelets, and shedding of GARP from their surfaces, combined with the fact that platelets are able to GARP dependently induce Treg, may be a highly disadvantageous combination that negatively affects patient survival. However, future studies must be conducted to evaluate platelet counts and their GARP levels as potential predictive and prognostic biomarkers for immunotherapy response and disease progression in melanoma.

In patients with inflammatory diseases, GARP has begun to be evaluated as a potential biomarker. For example, in patients with primary biliary cholangitis, an autoimmune biliary disease, GARP was found to be upregulated on cholangiocytes and was also detected on biliary duct cells (85). Herein, GARP expression was increased in response to biliary salts and released under cholestatic conditions *via* apoptosis of cholangiocytes. Atopic dermatitis patients showed a significantly reduced surface expression of GARP on Treg (21). Furthermore, patients with psoriasis, who received systemic therapy, had higher frequencies of activated GARP^+^ Treg than patients, who were only treated with topical therapy (86). Research in mice revealed that there is an increased expression of GARP on B cells in murine models of lupus (58). Whether these findings can be translated into the human setting has to be investigated in future trials.

Taken together, it will be interesting to validate GARP, either in its soluble or in its cell-associated form, as a predictive and prognostic biomarker in patients with immune mediated diseases in future clinical trials and to investigate whether GARP could be used as an early indicator for therapy response.

### GARP as a Therapeutic Target

As described in *section 5, Cellular GARP*, GARP is expressed by a variety of cells in the TME and plays a prominent role in immunosuppression and cancer progression. Drugs that modulate the expression of GARP may be useful for the treatment of various disease indications. Enhancing GARP expression, and its accompanying immunosuppression, may be beneficial for the treatment of autoimmunity and transplant rejection, whereas downregulating GARP expression may aid in the improvement of anti-tumor immune responses (**Figure 1D**).

Herein, several strategies are possible. On one hand, increased and long-lasting expression of GARP on Treg could lead to GARP^+^ Treg, which are more potent in inhibiting the proliferation and function of conventional Teff. This approach of improving Treg efficiency could be used in the treatment of autoimmunity and allergic diseases in order to modulate and downregulate overshooting immune or inflammatory processes.

Inhibition of miRNA, through the use of so called antagomirs, might be one possibility to induce elevated and long lasting GARP expression in Treg (**Figures 1A, D**). Antagomirs are oligonucleotides complementary to the mature form of specific miRNA. They easily penetrate into cells, and they inhibit the activity of their target miRNA, both *in vitro* and *in vivo* (87). Thus, they could represent a novel class of therapeutic molecules, leading to a sustained expression of GARP on Treg. Of note, transfer of miR-142-3p into Treg impaired their suppressive function (88). Importantly, Krützfeld et al. showed that antagomirs can be utilized as a systemic treatment and are resistant to degradation by RNases following injection into an organism (87). This approach should be further evaluated in future studies. One example would be to improve the clinical efficacy of human adoptive Treg transfer by enhancing the suppressive function and/or stability of Treg in an autoimmune or GvHD setting. Furthermore, in case of cancer and metabolic disease, the application of antagomir loaded nanoparticles to target unfavorable miRNA has already been demonstrated in several mouse studies (89, 90), supporting the potential of antagomirs to be used in novel therapeutic approaches.

As demonstrated earlier, sGARP can have a beneficial effect in the induction of peripheral Treg and can help sustain Treg differentiation. Additionally, sGARP can inhibit Teff functions by reducing their proliferation and inflammatory cytokine production (18, 50). When sGARP was applied *in vivo*, it was shown to inhibit overshooting immune responses in a humanized mouse model of allergy (91) as well as of transplant rejection and in GvHD (49). This opens up further strategies in using sGARP as an immunomodulatory agent, such as in transplantation or autoimmune diseases (**Figures 1C, D**).

Furthermore, the simultaneous administration of sGARP and LTGF-β could also be a promising treatment approach (**Figures 1B-D**). Herein, GARP could bind LTGF-β and enhance its activation. Since integrins are necessary for the final activation step of TGF-β, this would minimize the risk of putative side effects of exaggerated TGF-β activation. This approach was already proposed by Fridrich et al. in 2016 (50). In more detail, they were able to show that even small doses of sGARP could greatly enhance the activation of LTGF-β. This could have implications for autoimmune diseases, as a low dose combination application of sGARP and LTGF-β may mitigate the potential side effects of high dosages of pre-activated TGF-β (50).

On the one hand, Treg cellular therapies have been shown to ameliorate autoimmune diseases, graft rejections, and GvHD (92, 93). Elevating GARP expression levels on Treg to increase and prolong their activation status or using sGARP to increase the number of Treg could contribute to the suppression of autoimmune responses by restoring T cell tolerance. On the other hand, targeting of GARP on activated Treg through the use of blocking antibodies could enhance anti-tumor immunity. Several studies have reported that targeting GARP provided protective immunity against melanoma and colon cancer and the depletion of GARP on platelets did not lead to changes in hemostasis and thrombosis (75, 94).

Currently, two therapeutic antibody products are in phase 1 clinical trials: ABBV-151 (NCT03821935) and DS-1055 (NCT04419532). Both antibodies aim for the reactivation of anti-tumor immunity by specifically inhibiting Treg in malignant solid tumors. However, they differ in their mechanism of action by several points.

ABBV-151 is a human IgG4 monoclonal antibody (mAb) that specifically binds to the GARP/LTGF-β1 complex to inhibit the release of mature TGF-β1 from LTGF-β1 (**Figures 1B, D**) (95). This results in a Treg specific blocking effect on functional TGF-β1 release. This leads to the inhibition of TGF-β1 signaling in Treg and Teff, which in turn decreases the suppressive effects by Treg and restores Teff functions in the TME. In a preclinical study, a mouse surrogate antibody that targeted the GARP/LTGF-β1 complex improved anti-tumor effects in a combination setting with an anti-mouse programmed cell death protein 1 (PD-1) antibody when compared with the anti–PD-1 treatment alone. However, the former antibody did not display anti-tumor activity when administered as a monotherapy alone in a CT26 tumor mouse model (96). The anti-tumor combination effects of the anti-GARP-TGF-β1 antibody did not require FcγR-mediated effector functions. In the current clinical study ABBV-151 is applied in parallel as a monotherapy and in combination with an anti-PD-1 antibody.

DS-1055a is an anti-GARP afucosylated human IgG1 mAb, that aims for the efficient depletion of GARP^+^ Treg *via* antibody-dependent cellular cytotoxicity (**Figures 1A, D**) (97). This prevents Treg-mediated suppression of immune effector cells and thereby results in the reactivation of anti-tumor activities in the TME. In preclinical settings, DS-1055a treatment resulted in the depletion of GARP^+^ Treg and increased Teff functions *in vitro* and exerted anti-tumor effects in HT-29 tumor bearing humanized mice. In addition, combined treatment of DS-1055a with an anti-PD-1 antibody yielded a combination effect since the proliferation of Teff increased in comparison to treatment with either agent alone.

GARP targeting antibodies can be described as a kind of Treg specific treatment approach. In immune cells of the TME, GARP expression is almost completely limited to activated Treg. Unlike other targeted Treg associated proteins, this specificity in GARP expression on activated Treg is considered to be critical for the recovery of intrinsic anti-tumor activities without affecting effector immune functions. It is important to await the results of the aforementioned phase 1 clinical trials to determine whether the inhibition of Treg function or the removal of Treg themselves from the TME can lead to the revival and activation of Teff from their immunosuppressive dormant state, and thus result in tumor eradication.

Since, at least in preclinical models, the effects of combined treatment with an anti-PD-1 antibody have been confirmed. There is a rationale that GARP targeting antibodies have the potential to improve insufficient immune responses and decrease resistance to immune checkpoint inhibitors (ICI), like PD-1, programmed cell death protein ligand 1 (PD-L1), and CTLA-4, in clinical settings. In addition, the fact that GARP is expressed only by activated — not resting Treg, suggests that the actions of these antibodies should mainly occur in the highly immunosuppressive TME and should hopefully not lead to an increase in immune-related adverse events when used in combination with ICI.

In this regard, it has been shown that antibody mediated blockade or deletion of GARP did not alter innate or adaptive immune responses (98). Therefore, antibody therapies targeting GARP represent promising approaches in order to restore anti-tumor immunity without severely impairing other immunological defenses.

## Discussion

GARP presents as a promising target molecule in different disease settings. Of note, it is tightly associated to TGF-β and its functions. TGF-β is a pleiotropic cytokine and as such, a key mediator of many, often opposing biological processes. In cancer, TGF-β is a double-edged sword. It exerts potent cytostatic and pro-apoptotic activities in early stages of disease, but it can also paradoxically favor EMT and metastasis at later stages of malignant transformation, thus shifting from tumor inhibition to tumor promotion during the progression of cancer. In addition, it modulates the proliferation and function of different immune cells necessary for building a potent anti-tumor immune response, making it a difficult target for anti-cancer therapies. Therefore, due to this lack of specificity (in targeted function and cell type) resulting from the pleiotropic nature of TGF-β, combined with insufficient therapeutic efficacy, therapies directly targeting TGF-β have not entered the clinic for routine applications yet. In addition, clinical trials, which target TGF-β signaling, must be considered in combination with other therapies, including immune checkpoint blockade, chemotherapy, and radiotherapy.

One novel approach to address a particular function of TGF-β, is through the blockade of TGF-β1 activation by GARP expressing Treg using an anti-GARP:LTGF-β1 mAb. This would specifically prevent the release of TGF-β1 that is in complex with GARP. Whereas blocking of GARP:LTGF-β1 would decrease immunosuppression *via* Treg, another novel approach targets GARP itself.

This might delete important inhibitory cellular components of the TME, such as Treg and tumor cells. It can be argued that targeting and blocking GARP is a more promising upstream target, as GARP itself exerts a suppressive function on other cells as described above. Furthermore, cell surface complexation and soluble GARP have been shown to enhance TGF-β activation (50). Therefore, targeting GARP could lead to a simultaneous inhibition of TGF-β activation and function as well as the inhibition of the suppressive functions of GARP and sGARP. Whether an anti-GARP:TGF-β1 mAb is superior to using an anti-GARP mAb for blocking immune suppression in the TME will have to be analyzed in future studies. Both approaches have the potential to boost intrinsic anti-tumor activities and to show encouraging results in mouse models; however, a clinical validation is still awaited. Nevertheless, one challenge for the use of this therapeutic target could be the need for sufficient amounts of GARP on the surface of the mentioned cell types above. In addition, non-Treg side effects have to be taken into account, when using an anti-GARP antibody, as GARP is also expressed e.g. on activated platelets in peripheral blood (12). However, a GARP knockdown in platelets did not show any negative effects on hemostasis (75), and it also promoted anti-tumor immunity by inhibiting of TGF-β signaling in different cancer entities (94). Additionally, these findings point out a possible novel therapeutic approach in cancer based on the combination of GARP inhibition with platelet modulating agents, such as ticagrelor and aspirin (99).

It was shown by several groups (38) that GARP deficiency in Treg led to increased susceptibility to inflammatory diseases through the induction of immune dysregulation. Thus, GARP is somehow required to maintain immune homeostasis. It will be very interesting to investigate in more detail if and to what extend GARP alone contributes as an important molecule to Treg generation, stability, and function or whether GARP effects are solely modulated *via* regulation of TGF-β bioavailability.

In this context, the link between GARP and Foxp3 remains elusive. On the one hand, several publications support the idea that GARP and Foxp3 expression is independent of one and other (8, 36, 37, 46). On the other hand, it has been described that Treg specific transcription of LRRC32 is Foxp3 mediated, resulting from the synergistic interaction of Foxp3 with NFAT (5). Additionally, it has been suggested that there is a mutual dependency of Foxp3 and GARP expression, which influence Treg suppressive function. Future studies must be conducted to better understand the interdependence of GARP and Foxp3 expression in different cell types.

In most studies, GARP has been described as a surface molecule involved in the processing and maturation of TGF-β. Nevertheless, there are some reports showing cytoplasmatic and nuclear GARP expression as well (16). However, the function of this intracellular GARP and the significance of its intracellular localization need to be further characterized in future studies. These studies will help to clarify if GARP does have the same function on Treg, on platelets, and in cancer cells in means of the above-described functions, including suppressive capacity, proliferation, and therapy resistance.

Besides being a relevant therapeutic target, the use of GARP as a prognostic and predictive biomarker should be transferred into clinical routine. Quantification of GARP on peripheral blood cells, in serum, and on tumor tissue could potentially be used to reflect the immunosuppressive burden present in tumor patients. Up until now, there has been only little correlation between GARP expression and clinical tumor stages of patients. To gain more insight and to define a potential application of GARP as a biomarker, future studies should quantify GARP levels on blood cells, in serum, and on tumor tissues. Ideally, this should be performed with the use of multiplex approaches (100) in order to investigate in more detail the distribution and complex spatial interactions of GARP^+^ cells in the TME. These results can then be correlated back to clinical data.

Most data up until now has been generated from *in vitro* and *in vivo* (in mice) experimental models. There is an urgent need to translate and validate these results in the human system through clinical trials, as the findings of GARP (i.e. regulation) seem to be similar — but not identical to the murine setting.

## Conclusion

GARP is a highly promising target molecule in diverse disease settings, and it is expressed by different cells and tissues that exert immunomodulatory functions. As such, it could serve as a relevant new biomarker in patients with immune related diseases, such as cancer and autoimmunity.

In addition, targeting of membrane GARP as well as the use of soluble GARP are attractive therapeutic approaches for the treatment of a wide variety of malignant, autoimmune, and inflammatory diseases. Of note, one cannot consider the contribution of GARP to the immunosuppressive function of Treg in the absence of its key partner: LTGF-β. Nevertheless, novel approaches are needed as LTGF-β is produced and expressed ubiquitously, whereas cellular GARP expression is much more restricted.

## Author Contributions

NZ and AT took the lead in structuring and writing the manuscript. ET and KS wrote some sections, modified, and reviewed the manuscript. BG prepared the figure, modified, and reviewed the manuscript. All authors contributed to the article and approved the submitted version.

## Funding

This work was supported by CRC1066, TP-B14 to AT, Wilhelm-Sander-Foundation (2020.132.2) to AT, Hiege-Stiftung against skin cancer (200504) to AT, and a DAAD One-Year Research Grant for Doctoral Candidates (57552339) to ET.

## Conflict of Interest

KS is employed by Daiichi Sankyo Co., Ltd.

The remaining authors declare that the research was conducted in the absence of any commercial or financial relationships that could be construed as a potential conflict of interest.

## Publisher’s Note

All claims expressed in this article are solely those of the authors and do not necessarily represent those of their affiliated organizations, or those of the publisher, the editors and the reviewers. Any product that may be evaluated in this article, or claim that may be made by its manufacturer, is not guaranteed or endorsed by the publisher.

## References

[1] Bopp T Becker C Klein M Klein-Hessling S Palmetshofer A Serfling E. Cyclic Adenosine Monophosphate Is a Key Component of Regulatory T Cell-Mediated Suppression. J Exp Med (2007) 204:1303–10. doi: 10.1084/jem.20062129 PMC2118605 17502663

[2] Ollendorff V Noguchi T deLapeyriere O Birnbaum D. The GARP Gene Encodes a New Member of the Family of Leucine-Rich Repeat-Containing Proteins. Cell Growth Differ (1994) 5:213–9. 8180135

[3] Roubin R Pizette S Ollendorff V Planche J Birnbaum D deLapeyriere O. Structure and Developmental Expression of Mouse Garp, a Gene Encoding a New Leucine-Rich Repeat-Containing Protein. Int J Dev Biol (1996) 40:545–55. 8840187

[4] Chan DV Somani A-K Young AB Massari JV Ohtola J Sugiyama H. Signal Peptide Cleavage is Essential for Surface Expression of a Regulatory T Cell Surface Protein, Leucine Rich Repeat Containing 32 (LRRC32). BMC Biochem (2011) 12:27. doi: 10.1186/1471-2091-12-27 21615933 PMC3127830

[5] Haupt S Söntgerath VS Leipe J Schulze-Koops H Skapenko A. Methylation of an Intragenic Alternative Promoter Regulates Transcription of GARP. Biochim Biophys Acta (2016) 1859:223–34. doi: 10.1016/j.bbagrm.2015.11.003 26584734

[6] Zhou Q Haupt S Prots I Thümmler K Kremmer E Lipsky PE. miR-142-3p Is Involved in CD25+ CD4 T Cell Proliferation by Targeting the Expression of Glycoprotein A Repetitions Predominant. J Immunol (2013) 190:6579–88. doi: 10.4049/jimmunol.1202993 23650616

[7] Tran DQ Andersson J Wang R Ramsey H Unutmaz D Shevach EM. GARP (LRRC32) is Essential for the Surface Expression of Latent TGF-Beta on Platelets and Activated FOXP3+ Regulatory T Cells. Proc Natl Acad Sci USA (2009) 106:13445–50. doi: 10.1073/pnas.0901944106 PMC2726354 19651619

[8] Wang R Wan Q Kozhaya L Fujii H Unutmaz D. Identification of a Regulatory T Cell Specific Cell Surface Molecule That Mediates Suppressive Signals and Induces Foxp3 Expression. PLoS One (2008) 3:e2705. doi: 10.1371/journal.pone.0002705 18628982 PMC2442191

[9] Stanic B van de Veen W Wirz OF Rückert B Morita H Söllner S. IL-10-Overexpressing B Cells Regulate Innate and Adaptive Immune Responses. J Allergy Clin Immunol (2015) 135:771–80.e8. doi: 10.1016/j.jaci.2014.07.041 25240783

[10] Dedobbeleer O Stockis J van der Woning B Coulie PG Lucas S. Cutting Edge: Active TGF-β1 Released From GARP/TGF-β1 Complexes on the Surface of Stimulated Human B Lymphocytes Increases Class-Switch Recombination and Production of IgA. J Immunol (2017) 199:391–6. doi: 10.4049/jimmunol.1601882 PMC5502319 28607112

[11] Zhang M Pan X Fujiwara K Jurcak N Muth S Zhou J. Pancreatic Cancer Cells Render Tumor-Associated Macrophages Metabolically Reprogrammed by a GARP and DNA Methylation-Mediated Mechanism. Signal Transduct Target Ther (2021) 6:366. doi: 10.1038/s41392-021-00769-z 34711804 PMC8553927

[12] Zimmer N Krebs FK Zimmer S Mitzel-Rink H Kumm EJ Jurk K. Platelet-Derived GARP Induces Peripheral Regulatory T Cells-Potential Impact on T Cell Suppression in Patients With Melanoma-Associated Thrombocytosis. Cancers (Basel) (2020) 12. doi: 10.3390/cancers12123653 PMC7762193 33291452

[13] Xing H Liang C Xu X Sun H Ma X Jiang Z. Mesenchymal Stroma/Stem-Like Cells of GARP Knockdown Inhibits Cell Proliferation and Invasion of Mouse Colon Cancer Cells (MC38) Through Exosomes. J Cell Mol Med (2020) 24:13984–90. doi: 10.1111/jcmm.16008 PMC7753840 33155413

[14] Li Y Kim B-G Qian S Letterio JJ Fung JJ Lu L. Hepatic Stellate Cells Inhibit T Cells Through Active TGF-β1 From a Cell Surface-Bound Latent TGF-β1/GARP Complex. J Immunol (2015) 195:2648–56. doi: 10.4049/jimmunol.1500139 PMC4784714 26246140

[15] Probst-Kepper M Geffers R Kröger A Viegas N Erck C Hecht H-J. GARP: A Key Receptor Controlling FOXP3 in Human Regulatory T Cells. J Cell Mol Med (2009) 13:3343–57. doi: 10.1111/j.1582-4934.2009.00782.x PMC4516490 19453521

[16] Zimmer N Kim E Sprang B Leukel P Khafaji F Ringel F. GARP as an Immune Regulatory Molecule in the Tumor Microenvironment of Glioblastoma Multiforme. Int J Mol Sci (2019) 20. doi: 10.3390/ijms20153676 PMC6695992 31357555

[17] Carrillo-Gálvez AB Quintero JE Rodríguez R Menéndez ST Victoria González M Blanco-Lorenzo V. GARP Promotes the Proliferation and Therapeutic Resistance of Bone Sarcoma Cancer Cells Through the Activation of TGF-β. Cell Death Dis (2020) 11:985. doi: 10.1038/s41419-020-03197-z 33203838 PMC7673987

[18] Hahn SA Neuhoff A Landsberg J Schupp J Eberts D Leukel P. A Key Role of GARP in the Immune Suppressive Tumor Microenvironment. Oncotarget (2016) 7:42996–3009. doi: 10.18632/oncotarget.9598 PMC5190003 27248166

[19] Derycke MS Charbonneau B Preston CC Kalli KR Knutson KL Rider DN. Toward Understanding the Genetics of Regulatory T Cells in Ovarian Cancer. Oncoimmunology (2013) 2:e24535. doi: 10.4161/onci.24535 23894717 PMC3716752

[20] Ferreira MA Matheson MC Duffy DL Marks GB Hui J Le Souëf P. Identification of IL6R and Chromosome 11q13.5 as Risk Loci for Asthma. Lancet (2011) 378:1006–14. doi: 10.1016/S0140-6736(11)60874-X PMC3517659 21907864

[21] Manz J Rodríguez E ElSharawy A Oesau E-M Petersen B-S Baurecht H. Targeted Resequencing and Functional Testing Identifies Low-Frequency Missense Variants in the Gene Encoding GARP as Significant Contributors to Atopic Dermatitis Risk. J Invest Dermatol (2016) 136:2380–6. doi: 10.1016/j.jid.2016.07.009 27448748

[22] Nasrallah R Imianowski CJ Bossini-Castillo L Grant FM Dogan M Placek L. A Distal Enhancer at Risk Locus 11q13.5 Promotes Suppression of Colitis by Treg Cells. Nature (2020) 583:447–52. doi: 10.1038/s41586-020-2296-7 PMC7116706 32499651

[23] Wang R Zhu J Dong X Shi M Lu C Springer TA. GARP Regulates the Bioavailability and Activation of Tgfβ. Mol Biol Cell (2012) 23:1129–39. doi: 10.1091/mbc.E11-12-1018 PMC3302739 22278742

[24] Sun L Jin H Li H. GARP: A Surface Molecule of Regulatory T Cells That is Involved in the Regulatory Function and TGF-β Releasing. Oncotarget (2016) 7:42826–36. doi: 10.18632/oncotarget.8753 PMC5173174 27095576

[25] Choi G Na H Kuen D-S Kim B-S Chung Y. Autocrine TGF-β1 Maintains the Stability of Foxp3+ Regulatory T Cells *via* IL-12rβ2 Downregulation. Biomolecules (2020) 10. doi: 10.3390/biom10060819 PMC7356964 32471185

[26] Gordon KJ Blobe GC. Role of Transforming Growth Factor-Beta Superfamily Signaling Pathways in Human Disease. Biochim Biophys Acta (2008) 1782:197–228. doi: 10.1016/j.bbadis.2008.01.006 18313409

[27] Stockis J Colau D Coulie PG Lucas S. Membrane Protein GARP is a Receptor for Latent TGF-Beta on the Surface of Activated Human Treg. Eur J Immunol (2009) 39:3315–22. doi: 10.1002/eji.200939684 19750484

[28] Wu BX Li A Lei L Kaneko S Wallace C Li X. Glycoprotein A Repetitions Predominant (GARP) Positively Regulates Transforming Growth Factor (TGF) β3 and Is Essential for Mouse Palatogenesis. J Biol Chem (2017) 292:18091–7. doi: 10.1074/jbc.M117.797613 PMC5672034 28912269

[29] Stockis J Dedobbeleer O Lucas S. Role of GARP in the Activation of Latent TGF-β1. Mol Biosyst (2017) 13:1925–35. doi: 10.1039/c7mb00251c 28795730

[30] Metelli A Wu BX Fugle CW Rachidi S Sun S Zhang Y. Surface Expression of Tgfβ Docking Receptor GARP Promotes Oncogenesis and Immune Tolerance in Breast Cancer. Cancer Res (2016) 76:7106–17. doi: 10.1158/0008-5472.CAN-16-1456 PMC5504525 27913437

[31] Gauthy E Cuende J Stockis J Huygens C Lethé B Collet J-F. GARP Is Regulated by miRNAs and Controls Latent TGF-β1 Production by Human Regulatory T Cells. PLoS One (2013) 8:e76186. doi: 10.1371/journal.pone.0076186 24098777 PMC3787020

[32] Derynck R Budi EH. Specificity, Versatility, and Control of TGF-β Family Signaling. Sci Signal (2019) 12. doi: 10.1126/scisignal.aav5183 PMC6800142 30808818

[33] Annes JP Munger JS Rifkin DB. Making Sense of Latent TGFbeta Activation. J Cell Sci (2003) 116:217–24. doi: 10.1242/jcs.00229 12482908

[34] Edwards JP Thornton AM Shevach EM. Release of Active TGF-β1 From the Latent TGF-β1/GARP Complex on T Regulatory Cells is Mediated by Integrin β8. J Immunol (2014) 193:2843–9. doi: 10.4049/jimmunol.1401102 PMC4157079 25127859

[35] Liénart S Merceron R Vanderaa C Lambert F Colau D Stockis J. Structural Basis of Latent TGF-β1 Presentation and Activation by GARP on Human Regulatory T Cells. Science (2018) 362:952–6. doi: 10.1126/science.aau2909 30361387

[36] Wang R Kozhaya L Mercer F Khaitan A Fujii H Unutmaz D. Expression of GARP Selectively Identifies Activated Human FOXP3+ Regulatory T Cells. Proc Natl Acad Sci U S A (2009) 106:13439–44. doi: 10.1073/pnas.0901965106 PMC2726405 19666573

[37] Elkord E Abd Al Samid M Chaudhary B. Helios, and Not FoxP3, is the Marker of Activated Tregs Expressing GARP/LAP. Oncotarget (2015) 6:20026–36. doi: 10.18632/oncotarget.4771 PMC4652984 26343373

[38] Lehmkuhl P Gentz M Garcia de Otezya AC Grimbacher B Schulze-Koops H Skapenko A. Dysregulated Immunity in PID Patients With Low GARP Expression on Tregs Due to Mutations in LRRC32. Cell Mol Immunol (2021) 18:1677–91. doi: 10.1038/s41423-021-00701-z PMC8245512 34059789

[39] Tao R de Zoeten EF Ozkaynak E Chen C Wang L Porrett PM. Deacetylase Inhibition Promotes the Generation and Function of Regulatory T Cells. Nat Med (2007) 13:1299–307. doi: 10.1038/nm1652 17922010

[40] Pu M Chen J Tao Z Miao L Qi X Wang Y. Regulatory Network of miRNA on its Target: Coordination Between Transcriptional and Post-Transcriptional Regulation of Gene Expression. Cell Mol Life Sci (2019) 76:441–51. doi: 10.1007/s00018-018-2940-7 PMC11105547 30374521

[41] Liu C Li N Liu G. The Role of MicroRNAs in Regulatory T Cells. J Immunol Res (2020) 2020:3232061. doi: 10.1155/2020/3232061 32322593 PMC7154970

[42] Jebbawi F Fayyad-Kazan H Merimi M Lewalle P Verougstraete J-C Leo O. A microRNA Profile of Human CD8(+) Regulatory T Cells and Characterization of the Effects of microRNAs on Treg Cell-Associated Genes. J Transl Med (2014) 12:218. doi: 10.1186/s12967-014-0218-x 25090912 PMC4440568

[43] Zhang Y Wu BX Metelli A Thaxton JE Hong F Rachidi S. GP96 is a GARP Chaperone and Controls Regulatory T Cell Functions. J Clin Invest (2015) 125:859–69. doi: 10.1172/JCI79014 PMC4319419 25607841

[44] Duan X Iwanowycz S Ngoi S Hill M Zhao Q Liu B. Molecular Chaperone GRP94/GP96 in Cancers: Oncogenesis and Therapeutic Target. Front Oncol (2021) 11:629846. doi: 10.3389/fonc.2021.629846 33898309 PMC8062746

[45] Zhou AX Kozhaya L Fujii H Unutmaz D. GARP-TGF-β Complexes Negatively Regulate Regulatory T Cell Development and Maintenance of Peripheral CD4+ T Cells *In Vivo* . J Immunol (2013) 190:5057–64. doi: 10.4049/jimmunol.1300065 PMC3653571 23576681

[46] Edwards JP Fujii H Zhou AX Creemers J Unutmaz D Shevach EM. Regulation of the Expression of GARP/latent TGF-β1 Complexes on Mouse T Cells and Their Role in Regulatory T Cell and Th17 Differentiation. J Immunol (2013) 190:5506–15. doi: 10.4049/jimmunol.1300199 PMC3668701 23645881

[47] Qian W-J Monroe ME Liu T Jacobs JM Anderson GA Shen Y. Quantitative Proteome Analysis of Human Plasma Following *In Vivo* Lipopolysaccharide Administration Using 16O/18O Labeling and the Accurate Mass and Time Tag Approach. Mol Cell Proteomics (2005) 4:700–9. doi: 10.1074/mcp.M500045-MCP200 PMC1829297 15753121

[48] Metelli A Wu BX Riesenberg B Guglietta S Huck JD Mills C. Thrombin Contributes to Cancer Immune Evasion *via* Proteolysis of Platelet-Bound GARP to Activate LTGF-β. Sci Transl Med (2020) 12. doi: 10.1126/scitranslmed.aay4860 PMC7814995 31915300

[49] Hahn SA Stahl HF Becker C Correll A Schneider F-J Tuettenberg A. Soluble GARP has Potent Antiinflammatory and Immunomodulatory Impact on Human CD4^+^ T Cells. Blood (2013) 122:1182–91. doi: 10.1182/blood-2012-12-474478 23818544

[50] Fridrich S Hahn SA Linzmaier M Felten M Zwarg J Lennerz V. How Soluble GARP Enhances Tgfβ Activation. PLoS One (2016) 11:e0153290. doi: 10.1371/journal.pone.0153290 27054568 PMC4824412

[51] Sakaguchi S Yamaguchi T Nomura T Ono M. Regulatory T Cells and Immune Tolerance. Cell (2008) 133:775–87. doi: 10.1016/j.cell.2008.05.009 18510923

[52] Rappl G Pabst S Riemann D Schmidt A Wickenhauser C Schütte W. Regulatory T Cells With Reduced Repressor Capacities Are Extensively Amplified in Pulmonary Sarcoid Lesions and Sustain Granuloma Formation. Clin Immunol (2011) 140:71–83. doi: 10.1016/j.clim.2011.03.015 21482483

[53] Zhong Y Tang H Wang X Zeng Q Liu Y Zhao XI. Intranasal Immunization With Heat Shock Protein 60 Induces CD4(+) CD25(+) GARP(+) and Type 1 Regulatory T Cells and Inhibits Early Atherosclerosis. Clin Exp Immunol (2016) 183:452–68. doi: 10.1111/cei.12726 PMC4750594 26452441

[54] Noyan F Lee Y-S Zimmermann K Hardtke-Wolenski M Taubert R Warnecke G. Isolation of Human Antigen-Specific Regulatory T Cells With High Suppressive Function. Eur J Immunol (2014) 44:2592–602. doi: 10.1002/eji.201344381 24990119

[55] Eschborn M Weigmann B Reissig S Waisman A Saloga J Bellinghausen I. Activated Glycoprotein A Repetitions Predominant (GARP)-Expressing Regulatory T Cells Inhibit Allergen-Induced Intestinal Inflammation in Humanized Mice. J Allergy Clin Immunol (2015) 136:159–68. doi: 10.1016/j.jaci.2015.04.020 26145987

[56] Adriawan IR Atschekzei F Dittrich-Breiholz O Garantziotis P Hirsch S Risser LM. Novel Aspects of Regulatory T Cell Dysfunction as a Therapeutic Target in Giant Cell Arteritis. Ann Rheum Dis (2022) 81:124–31. doi: 10.1136/annrheumdis-2021-220955 PMC8762021 34583923

[57] Wang H Song H Pham AV Cooper LJ Schulze JJ Olek S. Human LAP+GARP+FOXP3+ Regulatory T Cells Attenuate Xenogeneic Graft Versus Host Disease. Theranostics (2019) 9:2315–24. doi: 10.7150/thno.30254 PMC6531299 31149046

[58] Wallace CH Wu BX Salem M Ansa-Addo EA Metelli A Sun S. B Lymphocytes Confer Immune Tolerance *via* Cell Surface GARP-TGF-β Complex. JCI Insight (2018) 3. doi: 10.1172/jci.insight.99863 PMC5928869 29618665

[59] Carrillo-Gálvez AB Gálvez-Peisl S González-Correa JE de Haro-Carrillo M Ayllón V Carmona-Sáez P. GARP is a Key Molecule for Mesenchymal Stromal Cell Responses to TGF-β and Fundamental to Control Mitochondrial ROS Levels. Stem Cells Transl Med (2020) 9:636–50. doi: 10.1002/sctm.19-0372 PMC7180295 32073751

[60] Carrillo-Galvez AB Cobo M Cuevas-Ocaña S Gutiérrez-Guerrero A Sánchez-Gilabert A Bongarzone P. Mesenchymal Stromal Cells Express GARP/LRRC32 on Their Surface: Effects on Their Biology and Immunomodulatory Capacity. Stem Cells (2015) 33:183–95. doi: 10.1002/stem.1821 PMC4309416 25182959

[61] Huong PT Nguyen LT Nguyen X-B Lee SK Bach D-H. The Role of Platelets in the Tumor-Microenvironment and the Drug Resistance of Cancer Cells. Cancers (Basel) (2019) 11. doi: 10.3390/cancers11020240 PMC6406993 30791448

[62] Yap ML McFadyen JD Wang X Ziegler M Chen Y-C Willcox A. Activated Platelets in the Tumor Microenvironment for Targeting of Antibody-Drug Conjugates to Tumors and Metastases. Theranostics (2019) 9:1154–69. doi: 10.7150/thno.29146 PMC6401411 30867822

[63] McAllister SS Weinberg RA. The Tumour-Induced Systemic Environment as a Critical Regulator of Cancer Progression and Metastasis. Nat Cell Biol (2014) 16:717–27. doi: 10.1038/ncb3015 PMC6220424 25082194

[64] Camerer E Qazi AA Duong DN Cornelissen I Advincula R Coughlin SR. Platelets, Protease-Activated Receptors, and Fibrinogen in Hematogenous Metastasis. Blood (2004) 104:397–401. doi: 10.1182/blood-2004-02-0434 15031212

[65] Palumbo JS Talmage KE Massari JV La Jeunesse CM Flick MJ Kombrinck KW. Tumor Cell-Associated Tissue Factor and Circulating Hemostatic Factors Cooperate to Increase Metastatic Potential Through Natural Killer Cell-Dependent and-Independent Mechanisms. Blood (2007) 110:133–41. doi: 10.1182/blood-2007-01-065995 PMC1896107 17371949

[66] Karpatkin S Pearlstein E Ambrogio C Coller BS. Role of Adhesive Proteins in Platelet Tumor Interaction *In Vitro* and Metastasis Formation *In Vivo* . J Clin Invest (1988) 81:1012–9. doi: 10.1172/JCI113411 PMC329625 3280598

[67] Stone RL Nick AM McNeish IA Balkwill F Han HD Bottsford-Miller J. Paraneoplastic Thrombocytosis in Ovarian Cancer. N Engl J Med (2012) 366:610–8. doi: 10.1056/NEJMoa1110352 PMC3296780 22335738

[68] Sierko E Wojtukiewicz MZ. Platelets and Angiogenesis in Malignancy. Semin Thromb Hemost (2004) 30:95–108. doi: 10.1055/s-2004-822974 15034801

[69] Schumacher D Strilic B Sivaraj KK Wettschureck N Offermanns S. Platelet-Derived Nucleotides Promote Tumor-Cell Transendothelial Migration and Metastasis *via* P2Y2 Receptor. Cancer Cell (2013) 24:130–7. doi: 10.1016/j.ccr.2013.05.008 23810565

[70] Leblanc R Peyruchaud O. Metastasis: New Functional Implications of Platelets and Megakaryocytes. Blood (2016) 128:24–31. doi: 10.1182/blood-2016-01-636399 27154188

[71] Cravioto-Villanueva A Luna-Perez P La Gutierrez-de Barrera M Martinez-Gómez H Maffuz A Rojas-Garcia P. Thrombocytosis as a Predictor of Distant Recurrence in Patients With Rectal Cancer. Arch Med Res (2012) 43:305–11. doi: 10.1016/j.arcmed.2012.06.008 22727694

[72] Honn KV Tang D. Hemostasis and Malignancy: An Overview. Cancer Metastasis Rev (1992) 11:223–6. doi: 10.1007/BF01307178 1423814

[73] Kim E-S Kim M-S Moon A. Transforming Growth Factor (TGF)-Beta in Conjunction With H-Ras Activation Promotes Malignant Progression of MCF10A Breast Epithelial Cells. Cytokine (2005) 29:84–91. doi: 10.1016/j.cyto.2004.10.001 15598443

[74] Guo S-W Du Y Liu X. Platelet-Derived TGF-β1 Mediates the Down-Modulation of NKG2D Expression and may be Responsible for Impaired Natural Killer (NK) Cytotoxicity in Women With Endometriosis. Hum Reprod (2016) 31:1462–74. doi: 10.1093/humrep/dew057 27130956

[75] Vermeersch E Denorme F Maes W de Meyer SF Vanhoorelbeke K Edwards J. The Role of Platelet and Endothelial GARP in Thrombosis and Hemostasis. PLoS One (2017) 12:e0173329. doi: 10.1371/journal.pone.0173329 28278197 PMC5344406

[76] Szepetowski P Ollendorff V Grosgeorge J Courseaux A Birnbaum D Theillet C. DNA Amplification at 11q13.5-Q14 in Human Breast Cancer. Oncogene (1992) 7:2513–7. 1461654

[77] Jin H Sun L Tang L Yu W Li H. Expression of GARP Is Increased in Tumor-Infiltrating Regulatory T Cells and Is Correlated to Clinicopathology of Lung Cancer Patients. Front Immunol (2017) 8:138. doi: 10.3389/fimmu.2017.00138 28261204 PMC5306210

[78] Zhang L Xu J Zhang X Zhang Y Wang L Huang X. The Role of Tumoral FOXP3 on Cell Proliferation, Migration, and Invasion in Gastric Cancer. Cell Physiol Biochem (2017) 42:1739–54. doi: 10.1159/000479442 28743116

[79] Karanikas V Speletas M Zamanakou M Kalala F Loules G Kerenidi T. Foxp3 Expression in Human Cancer Cells. J Transl Med (2008) 6:19. doi: 10.1186/1479-5876-6-19 18430198 PMC2386447

[80] Li R Sun J Yang F Sun Y Wu X Zhou Q. Effect of GARP on Osteogenic Differentiation of Bone Marrow Mesenchymal Stem Cells *via* the Regulation of Tgfβ1 *In Vitro* . PeerJ (2019) 7:e6993. doi: 10.7717/peerj.6993 31198639 PMC6535220

[81] Krebs FK Trzeciak ER Zimmer S Özistanbullu D Mitzel-Rink H Meissner M. Immune Signature as Predictive Marker for Response to Checkpoint Inhibitor Immunotherapy and Overall Survival in Melanoma. Cancer Med (2021) 10:1562–75. doi: 10.1002/cam4.3710 PMC7940230 33449393

[82] Kalathil S Lugade AA Miller A Iyer R Thanavala Y. Higher Frequencies of GARP(+)CTLA-4(+)Foxp3(+) T Regulatory Cells and Myeloid-Derived Suppressor Cells in Hepatocellular Carcinoma Patients are Associated With Impaired T-Cell Functionality. Cancer Res (2013) 73:2435–44. doi: 10.1158/0008-5472.CAN-12-3381 PMC3645275 23423978

[83] Jiang S Zhang Y Zhang X Lu B Sun P Wu Q. GARP Correlates With Tumor-Infiltrating T-Cells and Predicts the Outcome of Gastric Cancer. Front Immunol (2021) 12:660397. doi: 10.3389/fimmu.2021.660397 34421887 PMC8378229

[84] Erpenbeck L Schön MP. Deadly Allies: The Fatal Interplay Between Platelets and Metastasizing Cancer Cells. Blood (2010) 115:3427–36. doi: 10.1182/blood-2009-10-247296 PMC2867258 20194899

[85] Bombaci M Pesce E Torri A Carpi D Crosti M Lanzafame M. Novel Biomarkers for Primary Biliary Cholangitis to Improve Diagnosis and Understand Underlying Regulatory Mechanisms. Liver Int (2019) 39:2124–35. doi: 10.1111/liv.14128 31033124

[86] Wegner J Krebs FK Tuettenberg A von Stebut E. Treg Activation Status Depends on Psoriasis Therapy Regime. J Dtsch Dermatol Ges (2020) 18:1481–4. doi: 10.1111/ddg.14368 33373123

[87] Krützfeldt J Rajewsky N Braich R Rajeev KG Tuschl T Manoharan M. Silencing of microRNAs *In Vivo* With 'Antagomirs'. Nature (2005) 438:685–9. doi: 10.1038/nature04303 16258535

[88] Huang B Zhao J Lei Z Shen S Li D Shen G-X. miR-142-3p Restricts cAMP Production in CD4+CD25- T Cells and CD4+CD25+ TREG Cells by Targeting AC9 mRNA. EMBO Rep (2009) 10:180–5. doi: 10.1038/embor.2008.224 PMC2637310 19098714

[89] Ha J Kim M Lee Y Lee M. Intranasal Delivery of Self-Assembled Nanoparticles of Therapeutic Peptides and Antagomirs Elicits Anti-Tumor Effects in an Intracranial Glioblastoma Model. Nanoscale (2021) 13:14745–59. doi: 10.1039/d1nr03455c 34474460

[90] Tao Y Xu S Wang J Xu L Zhang C Chen K. Delivery of microRNA-33 Antagomirs by Mesoporous Silica Nanoparticles to Ameliorate Lipid Metabolic Disorders. Front Pharmacol (2020) 11:921. doi: 10.3389/fphar.2020.00921 32848718 PMC7419650

[91] Meyer-Martin H Hahn SA Beckert H Belz C Heinz A Jonuleit H. GARP Inhibits Allergic Airway Inflammation in a Humanized Mouse Model. Allergy (2016) 71:1274–83. doi: 10.1111/all.12883 26990894

[92] Schlöder J Berges C Tuettenberg A Jonuleit H. Novel Concept of CD4-Mediated Activation of Regulatory T Cells for the Treatment of Graft-Versus-Host Disease. Front Immunol (2017) 8:1495. doi: 10.3389/fimmu.2017.01495 29167672 PMC5682297

[93] Martin H Reuter S Dehzad N Heinz A Bellinghausen I Saloga J. CD4-Mediated Regulatory T-Cell Activation Inhibits the Development of Disease in a Humanized Mouse Model of Allergic Airway Disease. J Allergy Clin Immunol (2012) 129:521–8,528.e1-7. doi: 10.1016/j.jaci.2011.09.038 22078574

[94] Rachidi S Metelli A Riesenberg B Wu BX Nelson MH Wallace C. Platelets Subvert T Cell Immunity Against Cancer *via* GARP-Tgfβ Axis. Sci Immunol (2017) 2. doi: 10.1126/sciimmunol.aai7911 PMC5539882 28763790

[95] Powderly J Shimizu T LoRusso P Razak A Miller K Balar A. Abstract CT207: Phase 1 First-in-Human Study of ABBV-151 as Monotherapy or in Combination With Budigalimab in Patients With Locally Advanced or Metastatic Solid Tumors. Cancer Res (2021) 81:CT207–7. doi: 10.1158/1538-7445.AM2021-CT207

[96] de Streel G Bertrand C Chalon N Liénart S Bricard O Lecomte S. Selective Inhibition of TGF-β1 Produced by GARP-Expressing Tregs Overcomes Resistance to PD-1/PD-L1 Blockade in Cancer. Nat Commun (2020) 11:4545. doi: 10.1038/s41467-020-17811-3 32917858 PMC7486376

[97] Satoh K Kobayashi Y Fujimaki K Hayashi S Ishida S Sugiyama D. Novel Anti-GARP Antibody DS-1055a Augments Anti-Tumor Immunity by Depleting Highly Suppressive GARP+ Regulatory T Cells. Int Immunol (2021) 33:435–46. doi: 10.1093/intimm/dxab027 34235533

[98] Gaignage M Zhang X Stockis J Dedobbeleer O Michiels C Cochez P. Blocking GARP-Mediated Activation of TGF-β1 did Not Alter Innate or Adaptive Immune Responses to Bacterial Infection or Protein Immunization in Mice. Cancer Immunol Immunother (2022). doi: 10.1007/s00262-021-03119-8 PMC9294018 34973084

[99] Cho MS Gonzalez-Pagan O Court Pinto K Sood A Afshar-Kharghan V. The Inhibition of Platelets Restore Anti-Tumor Immune Response to Ovarian Cancer and Its Therapeutic Implication. Blood (2018) 132:3698. doi: 10.1182/blood-2018-99-116530

[100] Schupp J Christians A Zimmer N Gleue L Jonuleit H Helm M. In-Depth Immune-Oncology Studies of the Tumor Microenvironment in a Humanized Melanoma Mouse Model. Int J Mol Sci (2021) 22. doi: 10.3390/ijms22031011 PMC7864015 33498319

